# The Doctor’s Bill and the Governor’s Veto

**Published:** 1891-11

**Authors:** 


### THE DOCTOR'S BILL AND THE GOVERNOR'S VETO.

---

We are very pleased to learn, just as we go to press, that that unjust and foolish measure known as the "Drunken Doctor's Bill," which passed both houses of the horny-handed Legislature, just adjourned, has received the Governor's veto. There were those among these rural Solons who were seized with such an intemperate zeal for temperance that they wished to make it a misdemeanor for a physician to become intoxicated.

With the printer demanding "copy," we have not the time to write a lengthy editorial. We have expressed our mind on this matter already. (See August number of JOURNAL.) The passage of this iniquitous bill was a reflection on one of the best and noblest classes of society, and we have been surprised that certain physicians in the Legislature gave it their support. We are glad that Governor Northen has put his disapproval on this narrow-minded and high-handed piece of legislation.

The Governor thus states his position:

"In this policy of legislation I cannot concur. Drunkenness on the part of practicing physicians and prescription clerks is reprehensible, and ought to be suppressed; but if it is a crime for them to be intoxicated, it ought to be a crime for others who get in like condition; and if it is no crime for others, it ought not to be a crime for them. It is the act of drunkenness that would be punished if this act were a law, and the acts arising from that condition; and if drunkenness is the *gravamen* of the offense all persons should come under the same law. If not all, then none."

From this wise and broad view of the matter at home, let us turn to the criticisms which come from abroad.

The *N. Y. Medical Record* says:

"The Georgia law is an insult to the profession of the State, because its existence implies a serious and preponderant degree of intemperance among medical men. It is in addition a form of special and class legislation of the worst character. Under it, a doctor who has led a sober and useful life for a long time may, as the result of a single indiscretion, have his career ruined and his means of subsistence taken away. But the lawyer or editor or farmer can stay drunk for a year with relative impunity. If such a statute, therefore, really exists, the physicians of Georgia should see to it that it is repealed, or be convicted of possessing neither manhood nor seif-respect."

---

HYDROPHOBIA AND SYNTAX.—The good citizens of Connecticut have been considerably alarmed at the prevalence of hydrophobia among the dogs of the State. The following remarkable notice appeared in the columns of a Farmington journal: "By notice elsewhere it will be see that any persons owning a dog in the town of Farmington are notified that they must be properly muzzled."—*Medical Record.*

Which reminds us of the notice the Georgia farmer posted on his premises: "If any man's or woman's cow or bull gits in this yere pastur, his or her tail will be cut off, as the case may be."

---

DR. W. A. CROW, of Atlanta, is now pursuing a post-graduate course at the New York Polyclinic and Hospital.

DR. A. B. MCRAE, of Seville, Georgia, is also at the Polyclinic.

